# Breastfeeding a small for gestational age infant, complicated by maternal gestational diabetes: a case report

**DOI:** 10.1186/s12884-019-2366-8

**Published:** 2019-06-21

**Authors:** Alexandra D. George, Melvin C. L. Gay, Mary E. Wlodek, Donna T. Geddes

**Affiliations:** 1 School of Molecular Sciences, The University of Western Australia, Crawley, Perth, Western Australia 6009 Australia; 2 Department of Physiology, School of Biomedical Sciences, Faculty of Medicine, Dentistry and Health Sciences, The University of Melbourne, Parkville, Victoria 3010 Australia

**Keywords:** Breastfeeding, Lactation, Human milk, Small for gestational age, Placental insufficiency, Gestational diabetes mellitus, Case report

## Abstract

**Background:**

Small for gestational age (SGA) infants are those born small for their gestational age, with weight below the 10th percentile. Not only do SGA infants suffer growth issues after birth, they have elevated risk for the development of metabolic and cardiovascular diseases later in life. Current research has suggested that in cases of SGA infants, maternal milk and breastfeeding are not affected.

**Case summary:**

The mother of an SGA infant was diagnosed with placental insufficiency and Gestational Diabetes Mellitus (GDM) during her pregnancy. The infant was born term, at 38 weeks 3 days, and SGA. The mother had a low milk supply and her milk composition differed from reference values such that the daily infant intake provided less than 50% of the required energy intake at 3 months.

**Conclusion:**

In cases of SGA and/or GDM, maternal milk quality and quantity may be compromised. This requires follow-up in order to reduce the disease risk for SGA infants and the corresponding public health implications.

## Background

Small for gestational age (SGA) infants account for approximately 10% of live births in developed countries and are those born below the 10th percentile for their age, as a result of in utero growth restriction [1]. Factors causing growth restriction include maternal disease, stress, smoking, malnutrition and placental insufficiency. While maternal malnutrition is the primary cause of growth restriction in low-income countries, placental insufficiency is the most common cause in high-income countries [2]. Placental insufficiency causes reduced blood, nutrient and oxygen supply to the developing fetus, consequently restricting fetal growth. Another common pregnancy complication resulting in growth restriction is Gestational Diabetes Mellitus (GDM), however the metabolic dysfunction that occurs in GDM can also produce average or large for gestational age infants [3]. In utero growth plays a critical role in programming lifelong health and disease, therefore insults during this time that lead to altered growth could result in sex-specific increased risk of cardiovascular and metabolic diseases [4, 5]. These diseases, however, often require further insult (such as pregnancy, which places additional stress on the maternal adaptations to pregnancy) to reveal the dysfunction. This can also impact the next generation [6]. In order to minimise this programmed disease risk, the infant’s weight gain would ideally match their genetic growth trajectory. However 10 to 30% of SGA infants do not shift their weight trajectory upwards and continue to experience poor growth throughout childhood [7]. Conversely, the infant may experience very rapid weight gain, which is also associated with increased risk of obesity and metabolic diseases in later life [8, 9]. Human milk is the ideal nutrition for the infant, shown to promote optimum growth and thought to uniquely match the infant’s requirements [10]. The human milk macronutrient concentrations in healthy women breastfeeding healthy term infants are 35–50 g/L fat, 9–12 g/L protein, and 60–100 g/L lactose, with a minimum volume of 478 mL/day required [11, 12]. Animal studies have reported that infant growth restriction results in compromised maternal quantity and quality of milk [13]. This is in contrast to the few human studies that have reported no change in milk composition in cases of growth restriction [14–16]. These studies, however, used inappropriate sampling methods for fat analysis, collecting samples from one time point in the first 28 days post-partum, and did not consider the daily or monthly variation of fat [11, 17]. Further, they did not measure infant milk intake and therefore could not determine the macronutrient dosage the infant received. We present the first case study in which both the quantity and quality of human milk was compromised in a lactating mother who experienced placental insufficiency and GDM, and gave birth to an SGA infant.

## Case presentation

A 32-year-old Caucasian woman was recruited during her first pregnancy for a longitudinal breastfeeding research study (UWA Human Research Ethics, reference RA/4/20/4023). Timing of samples and measurements collected are outlined in Table 1.

**Table 1:** Timing schedule for collection of maternal milk samples, 24-h milk production, and infant anthropometric measurements

Month	Pre-feed milk sample	24-h maternal milk production	Infant anthropometric measurements
0			*
1	*		*
2	*		*
3		*	*
4	*		*
5	*		*
6	*		*
12			*

Prior to conception the woman had a healthy BMI of 19.8 and Crohn’s disease, medicated with Adalimumab. During pregnancy she was diagnosed with GDM and was otherwise healthy with no other reported cardiovascular or metabolic diseases. Overall, the woman reported having a healthy balanced diet, having no allergies, and being a non-smoker. Her infant was fed colostrum and she experienced breast fullness on day 4, coinciding with secretory activation. The mother was advised to pump as frequently as possible, in addition to breastfeeding, and had breastfeeding support at home, although she did not have breastfeeding support from healthcare providers until her infant was 4 months old. It is of interest that the mother herself was born SGA, 2100 g at 40 weeks.

**Fig. 1:**
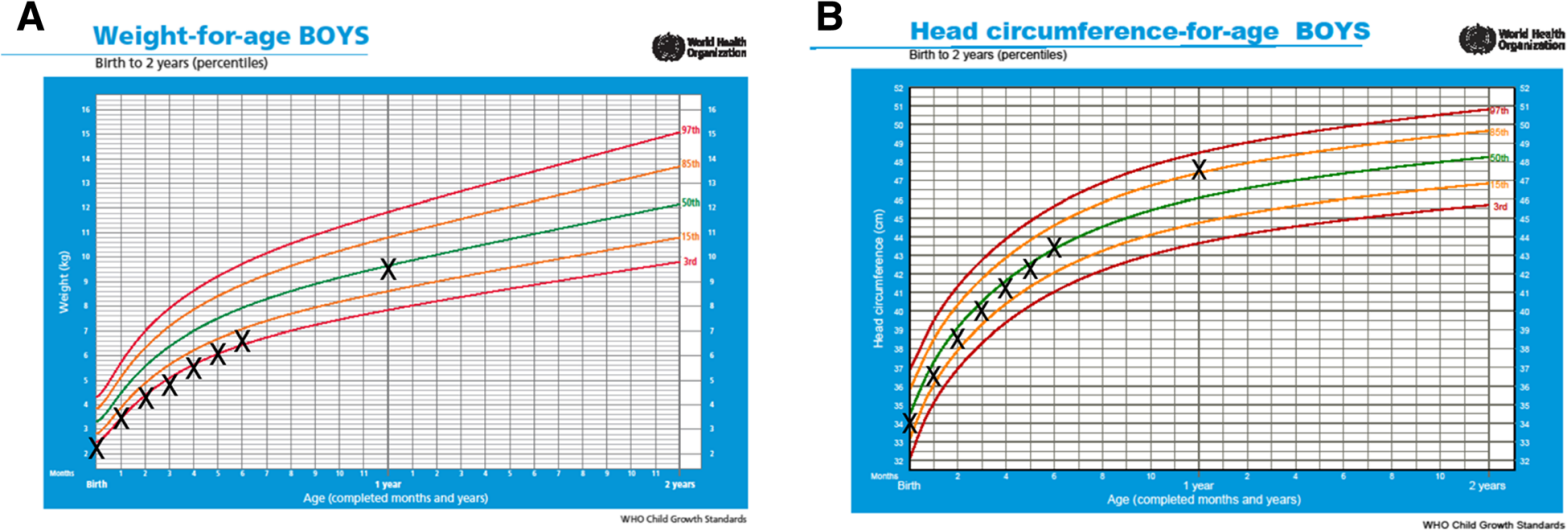
Weight (**a**) and head circumference (**b**) for SGA infant for first 12 months of life. The infant was exclusively breastfed for 3.5 months after which formula supplementation was initiated. Solid food was introduced after 5 months. Measurements are plotted on WHO growth charts with 97th, 85th, 50th, 15th and 3rd growth percentile curves indicated by the coloured lines

The male infant was delivered vaginally at 38 weeks and 3 days, after induction due to diagnosed placental insufficiency resulting in fetal growth restriction. The infant birth weight was 2390 g, and below the 3rd percentile. For the first 3.5 months of life, the exclusively breastfed infant was tracking along the 3rd percentile for growth, as shown in Fig. 1a. After 3.5 months, formula supplementation was initiated yet the infant growth continued along the 3rd percentile. In contrast, the infant was born with a head circumference of 34 cm (Fig. 1b), just below the 50th percentile, indicating head sparing as is characteristic of SGA infants. He was otherwise healthy, with no tongue tie or other oral abnormalities, and averaged 12 feeds per day (within the population range). Solids were introduced at 5 months of age. At 1 year, the infant weighed 9604 g with a head circumference of 47.8 cm, corresponding to the 50th and 85th percentile respectively.

### Milk production

As part of the longitudinal study, maternal milk production was measured at 3 months post-partum by weighing the infant before and after each feed for 24 h using an electronic scale [12]. A volume of 414 mL/day was produced, below the reference range (478–1298 mL/day). Infant milk intake for that day was 419 mL, of both breastfed and expressed milk. The breastfeeding profile falls within range for the number of breastfeeds and duration of breastfeed, but not for the average breastfeed volume from the left breast nor the milk volume that was breastfed and expressed (Table 2). A total daily dose of 60 mg Domperidone was initiated at 6 weeks post-partum, presumably to address issues of low milk supply, along with regular pumping after and between feeds, and continued up until 7 months of lactation, however she reported that her dosage decreased over this time.

**Table 2:** Maternal 24-h milk production profile at 3 months post-partum

	Case study			Population range
	Left	Right	Total	
Number of breastfeeds	6	6	12	6–18
Average breastfeed (mL)	12	37	49	30–135
Average breastfeed duration (min)	9	12		5–37
24 h milk intake breastfeeding (mL)	70	224	294	478–1298
Expressed milk (mL)	60	60		
Expressed milk intake (mL)			125	
Total infant milk intake (mL)			419	
Total milk produced in 24 h (mL)			414	

Milk production population reference ranges from Kent et al. [12]

### Milk composition

As part of the longitudinal study, monthly pre-feed samples were collected from birth to 6 months post-partum, and additional samples were also collected for every feed or expression during the 24-h milk production at 3 months [18]. Samples were collected by hand expression into 5 mL plastic vials and were stored frozen at − 20 °C for up to 48 h then transferred to − 80 °C laboratory freezer until analysis. The total fat concentration of the whole milk was measured using the creamatocrit method [19]. The protein concentration of skimmed milk was measured using the Bradford Protein Assay with human milk protein standards. The lactose concentration of skimmed milk was measured using an enzymatic spectrophotometric method [11]. Replicate analyses were carried out for all samples.

**Fig. 2:**
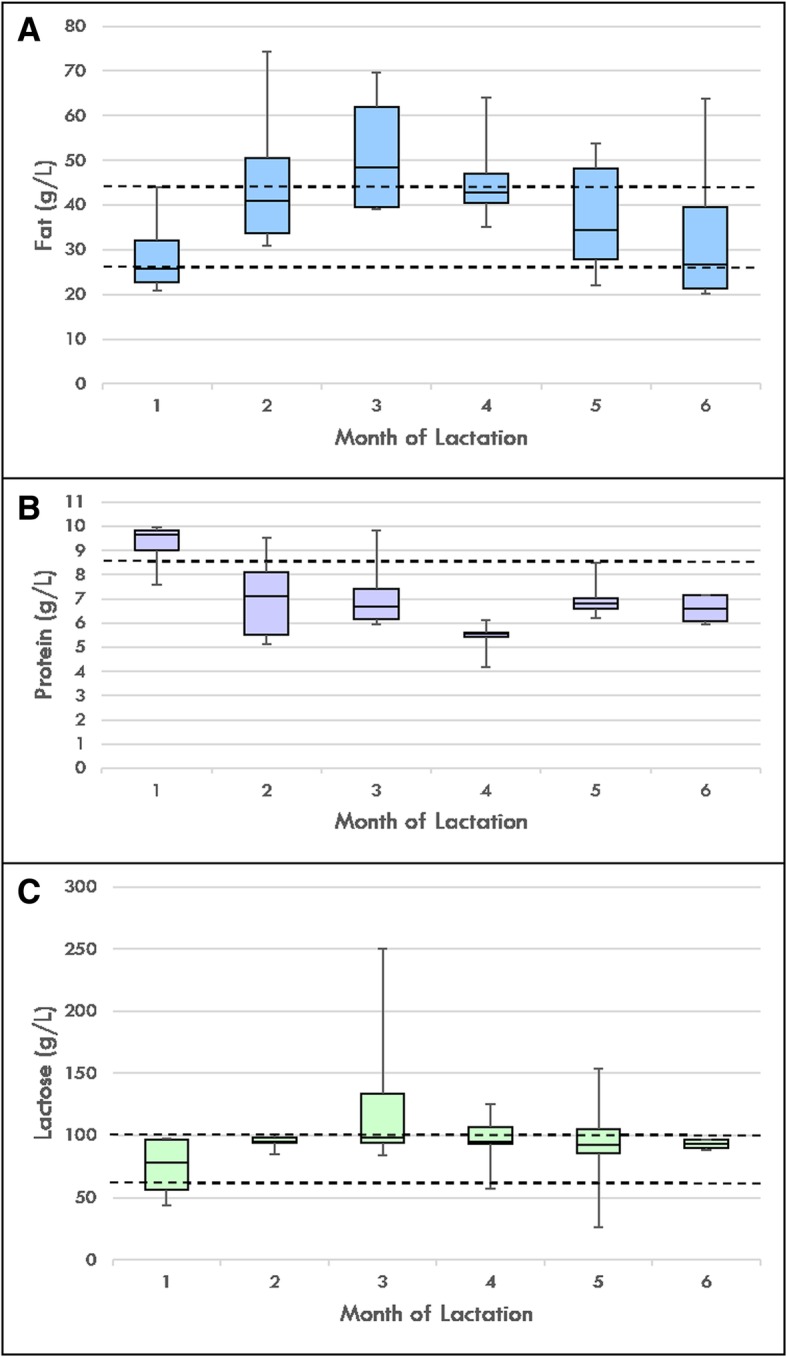
Total maternal milk fat (**a**), protein (**b**) and lactose (**c**) concentrations. Months 1 (*n* = 14), 2 (*n* = 6) and 3 (*n* = 14) exclusive breastfeeding. Months 4 (*n* = 6), 5 (*n* = 6) and 6 (*n* = 4) mixed breast and formula feeding, with solids introduced at 5 months. Dotted lines indicate macronutrient reference ranges for healthy term infants, from Mitoulas et al. [16]

The median fat concentration measured in 1, 5 and 6 months post-partum (25.8, 34.3 and 26.6 g/L, respectively) were below the reference range (36-50 g/L; Fig. 2a). All samples from 2 to 6 months post-partum (ranging from 4 to 8 g/L) had median protein concentrations below the reference range (9–12 g/L; Fig. 2b). The median lactose concentrations were within the reference range (60-100 g/L) at each month (Fig. 2c). References ranges were taken from those previously established in milk samples of healthy mothers breastfeeding healthy term infants [11].

### Infant energy intake

Infant total energy intake (both breastfed and expressed milk intake) at 3 months was calculated using infant milk volume intake and energy per gram of fat (37.7 kJ/g), protein (16.7 kJ/g) and lactose (16.7 kJ/g). Infant daily energy intake from fat, protein and lactose was approximately 595 kJ, 46 kJ and 537 kJ, respectively. The infant total daily energy intake was 1178 kJ/day, amounting to a low total energy dose from fat, protein and lactose per day (Table 3).

**Table 3:** Maternal milk macronutrient concentrations, dose and corresponding energy at 3 months post-partum

Macronutrient (Reference range, g/L)	Mean concentration (SD)	Daily dose (g/kg/day)	Energy (kJ/day)	Energy dose (kJ/kg/day)
Fat (35–50)	51.6 (13.2)	4.4	595	120
Protein (9–12)	6.6 (1.6)	0.6	46	9
Lactose (60–100)	76.7 (6.6)	6.5	537	108
Total energy intake			1178	237
Total estimated requirement^a^			2400	456

Macronutrient reference ranges from Mitoulas et al. [11]. ^a^Estimated requirements for a 3-month-old male infant

## Discussion and conclusions

In this case study we provide evidence that human milk quantity and quality are compromised in a lactating mother who had GDM and placental insufficiency, delivering an SGA infant. Maternal milk volume in this case was well below the average volume and below the reference range for successful breastfeeding women of healthy term infants [18]. Milk volume was not improved by the administration of a galactogogue, and was independent of the fact that the mother was feeding as frequently as mothers of healthy term infants, strongly suggesting that the deficiency in milk production was of an intrinsic nature. Although we cannot comment on mammary development and differentiation in this case, and it is not fully understood how pregnancy complications can affect milk production in women, SGA rat studies have shown both compromised quantity of milk delivered to the offspring and reduced mammary tissue [13]. No human studies have measured milk production in women of SGA infants, despite studies attributing a greater proportion of infant growth to milk volume than macronutrient composition [18, 20]. Low volume of milk produced by this mother in part accounts for the poor growth of the infant. Whilst the volume was near the lower limit of the range for term infants, males are not only more susceptible to impacts in pregnancy, such as growth restriction, they also require greater milk volumes than females [12, 21]. Despite the introduction of formula at 3.5 months the infant still maintained growth along the 3rd percentile, not increasing until after the introduction of solid food. This suggests either the infant did not receive enough formula and breast milk to improve growth or there is a time lapse for recovery from an extended period of undernutrition (pregnancy and 3 months post-partum). Undernutrition early in life carries as many risks as over nutrition, potentially affecting infant brain development, immunity and growth, and increasing disease risk in adulthood [22].

Interestingly, the macronutrient milk composition differed considerably from reference ranges for term breastfed infants in this SGA case, despite other studies failing to show differences [11, 14–16]. This is likely due to a combination of sampling limitations that exist in previous literature. Samples in previous SGA studies were collected between days 0 and 28 post-partum, mid-feed or from an entire breast emptying, and only collected at one time point during the day. Human milk fat is known to be highly variable, increasing through a feed and throughout the day, therefore the samples collected in previous studies were not representative of the nutrients that the infant ingested for each feed [23]. We have considered these variations by sampling pre-feed and multiple times each day over the 6 months period that we collected samples. Furthermore, the volume produced by the mothers in these previous studies was not considered, therefore measuring the mother’s 24-h production at 3 months in this case study was valuable in calculating the dose that the infant was receiving.

In this case study the milk protein concentration was low. Protein is required for lean tissue accretion so this may predispose the infant to altered body composition now and later in life. Formula fed infants have a higher protein intake than breast milk fed infants, and this is thought to be a primary contributor to fat free mass accretion, suggesting that the low protein found in this study could result in compromised fat free mass accretion. Specifically, high intake of the protein casein has been shown to be associated with lower fat free mass and higher fat mass, although casein was not measured in this case study [24]. Fat content was low in this case study and as it contributes to approximately 50% of the infant energy from human milk this would also impact the infant’s growth and development. Fat is essential for infant neural and retinal development, with human milk fat contributing to neurodevelopmental changes in the first few months of life and fatty acid accumulation in the brain occurring in the first 24 months of life, particularly the fatty acids docosahexaenoic acid and arachidonic acid [25, 26]. Lactose is the principal carbohydrate in human milk, providing approximately 40% of the total energy the infant receives from milk, and remains the most constant throughout lactation [27]. The average lactose concentrations in this case did not lie outside the reference range, which is in contrast to one study which found total carbohydrates to be significantly lower than those in the milk provided to average- and large-for-gestational age infants, although this was not considered a clinically significant reduction in carbohydrates for the infant [16]. However, when using the measured concentrations of fat, protein and lactose and the milk volume we found that the infant was receiving only 49% of its daily required energy. This is explains both the low weight of the infant and the lack of catch up growth without adequate supplementation.

It is of note that the mother of this infant was also born SGA, therefore had an increased risk of adult diseases and pregnancy complications. The fact that she developed GDM and delivered an SGA infant supports the hypothesis of transgenerational disease transmission [28]. Furthermore, an animal model of uteroplacental insufficiency has reported that the females who are born small subsequently develop glucose intolerance indicative of GDM [29]. The exact pathways to explain this result are complicated due to the mother experiencing not only placental insufficiency but GDM. Mammary ductal growth and alveolar development requires a number of hormones, including estrogen and progesterone that are produced by the placenta, therefore placental insufficiency may have contributed to a reduction of these hormones leading to reduced mammary development as reported in rat models [13]. Further, GDM causes hyperglycaemia due to reduced insulin sensitivity, which has been shown to predict poor milk supply in women attempting to exclusively breastfeed [30]. Insulin has essential roles in the regulation of the developing mammary gland, therefore insulin resistance may have hindered lactation by reducing mammary development, impairing lactocyte differentiation, delaying the initiation of lactation and impacting the rate of milk production [31–34].

Current research does not adequately characterise the human milk of mothers with SGA infants, in this case report we describe altered milk composition and volume, indicating that placental insufficiency (with GDM) in humans could impact lactation. Further investigation is warranted in humans, with a more comprehensive study design for pregnancy complications such as being born small for gestational age or the mother having GDM (including maternal glucose tolerance testing, ultrasound, placenta histology, and collection of other clinical data). Future human milk research in this area is essential in order to determine whether pregnancy complications, such as growth restriction, placental insufficiency and GDM, are linked to poor lactation outcomes.

## Data Availability

The datasets generated and/or analysed during the current study are not publicly available in order to further preserve the confidentiality of the participant. Data sets are available from the corresponding author on request.

## Abbreviations

GDM: Gestational Diabetes Mellitus
SGA: Small for Gestational Age
